# A Lightweight Vehicle-Pedestrian Detection Algorithm Based on Attention Mechanism in Traffic Scenarios

**DOI:** 10.3390/s22218480

**Published:** 2022-11-04

**Authors:** Yong Zhang, Aibo Zhou, Fengkui Zhao, Haixiao Wu

**Affiliations:** 1 School of Automotive and Traffic Engineering, Nanjing Forestry University, Nanjing 210037, China; 2 School of Energy and Power Engineering, Nanjing University of Aeronautics and Astronautics, Nanjing 210016, China

**Keywords:** autonomous driving, object detection, YOLOv4, MobileNetv2, coordinate attention mechanism, multi-scale feature fusion

## Abstract

Object detection is a critical technology of environmental perception for autonomous driving vehicle. The Convolutional Neural Network has gradually become a powerful tool in the field of vehicle detection because of its powerful ability of feature extraction. In aiming to reach the balance between speed and accuracy of detection in complex traffic scenarios, this paper proposes an improved lightweight and high-performance vehicle–pedestrian detection algorithm based on the YOLOv4. Firstly, the backbone network CSPDarknet53 is replaced by MobileNetv2 to reduce the number of parameters and raise the capability of feature extraction. Secondly, the method of multi-scale feature fusion is used to realize the information interaction among different feature layers. Finally, a coordinate attention mechanism is added to focus on the region of interest in the image by way of weight adjustment. The experimental results show that this improved model has a great performance in vehicle–pedestrian detection in traffic scenarios. Experimental results on PASCAL VOC datasets show that the improved model’s *mAP* is 85.79% and speed is 35FPS, which has an increase of 4.31% and 16.7% compared to YOLOv4. Furthermore, the improved YOLOv4 model maintains a great balance between detection accuracy and speed on different datasets, indicating that it can be applied to vehicle–pedestrian detection in traffic scenarios.

## 1. Introduction

In recent years, with the rapid development of artificial intelligence technology, deep learning [1] has been widely applied in various fields, including autonomous driving. The rapid development of autonomous technology has a positive significance for reducing the number of traffic accidents and improving the efficiency of transportation. There are more and more challenges for the environmental perception of autonomous driving vehicles due to the complex traffic environment. At present, the remarkable progress of computer vision and computation tools have provided theoretical and technical support for autonomous environmental perception [2]. Therefore, vision-based object detection is a key means of autonomous environmental perception [3,4], and its detection performances, such as accuracy, speed, efficiency, and robustness, are very important for the detection of vehicles, pedestrians, and obstacles in traffic scenes [5,6].

This paper further explores the framework of a low-consumption, high-precision, and lightweight vehicle–pedestrian detection model to meet the objective requirements of the diversity of targets and objection speed in traffic scenes. In summary, some structures are optimized on the basis of the original YOLOv4 [7,8,9] to make the model more suitable for the particularity of the traffic scene. The main work can be concluded as follows.

(1) To simplify the backbone network and improve the speed of vehicle–pedestrian detection obviously, the original backbone network CSPDarknet53 is replaced with MobileNetv2 embedded with Depthwise Separable Convolution [10].
(2) The feature pyramid structure is optimized by means of multi-scale feature fusion to realize the interaction of feature information among different feature layers.
(3) The coordinate attention mechanism is applied to focus on the region of interest in the image by adjusting the weights so as to enhance the feature extraction capability of the model.

## 2. Related Work

### 2.1. Object Detection

At present, the algorithms of object detection based on deep learning are roughly divided into two frameworks: two-stage and one-stage. The former generates the target candidate region through the Convolutional Neural Network, then uses the classifier to predict the categories and positions in the target candidate region. The two-stage detection algorithm is superior in detection accuracy; such algorithms mainly include Region-Convolutional Neural Network (R-CNN) series, Spatial Pyramid Pooling in deep convolutional networks for visual recognition (SPP-Net), Fast R-CNN [11], etc. However, this type of algorithm has many shortcomings, such as low operating efficiency and poor real-time performance. The latter is mainly represented by the You Only Look Once (YOLO) [12] series and the Single Short multi-box Detector (SSD) [13] series. The one-stage algorithm regards the detection task as a regression problem and directly gets the object category and position information, which is end-to-end object detection. Therefore, it has simple detection steps, high detection efficiency, and great real-time performance, which is especially suitable for embedded devices as a lightweight detection network. Alexey et al. proposed the YOLOv4 algorithm based on the previous YOLO series, which integrated many popular techniques, such as Mosaic data enhancement, CIOU_Loss, and partial residual structure. Since then, YOLOv4-Tiny has been proposed as a lightweight version of YOLOv4, which greatly simplifies the backbone of the feature extraction network. Chen et al. [14] proposed the YOLOF network, which used an expanded encoder and balanced matching to replace multi-scale feature fusion. The strategy shows that the detection speed can be improved to a certain extent without sacrificing the detection performance. Zheng et al. proposed the YOLOX network, which creatively switches the detection head into an anchor-free mode and decouples it into independent feature channels to optimize the model convergence speed and accuracy. Peng et al. [15] used YOLOv4 to extract vehicle features adaptively instead of subjective selection. Ma et al. [16] optimized the backbone network and feature pyramid fusion network based on YOLOv4-tiny, fully extracted global and local features, and solved the problem of insufficient accuracy of the original network due to lack of illumination and target occlusion.

With the rapid expansion of Convolutional Neural Networks and digital graphic processing techniques in the past few decades, the number of parameters and computation load of huge neural networks are becoming new challenges for object detection, which need the help of the GPU’s strong computing power. The problem that a large neural network is not suitable to use on mobile devices prompts the rapid development of lightweight networks. A series of models have emerged, such as SqueezeNet, ShuffleNet [17], Xception [18], and MobileNet [19,20], which make it possible for embedded and other edge devices to run deep learning models directly. Zhou et al. [5] proposed the MobileYOLO model, which used the mobilenet to effectively reduce model parameters. Zhao et al. [21] proposed an improved YOLO model to adapt to real-time detection in a special natural geographical environment. The improved model used the ideal of lightweight networks, transfer learning, and pretrained weights on large datasets. Li et al. [22] proposed a fruit detection model based on YOLOv3, which replaces the darknet53 network with MobileNetv2 to realize the lightweight deployment of picking machines. Li et al. [23] proposed YOLO-V3-Tiny-MobileNet, which solved the problem of insufficient feature extraction ability to achieve the improvement in parameters number, mean average precision, and detection performance.

### 2.2. Object Detection Based on Attention Mechanism

Similar to the human brain, the computer vision uses Attention Mechanism to analyze and process some complex application scenes efficiently and quickly. The attention mechanism [24] in the field of computer vision is capable of extracting the region of interest in the image more effectively and ignoring the irrelevant parts by adjusting the weights. Hu et al. [25] proposed the concept of channel attention and pioneered the SENet attention framework, the core of which is to use a squeeze-and-excitation module (SE) to collect global information better and capture the feature channels interrelationships that effectively improve the features expression ability. Wang et al. [26] proposed efficient channel attention (ECA) to directly establish a corresponding relationship between the input value and the weight vector due to the reduction in the number of feature channels. They use one-dimensional convolution instead of dimensionality reduction to aggregate global spatial information and model the efficient excitation of cross-channel interactions. Zhang et al. [27] proposed a context encoding module (CEM) on the basis of SENet to make full use of global scene context information for semantic segmentation. Woo et al. [28] proposed a lightweight and general Convolutional Block Attention Module (CBAM). This approach infers attention maps along both channel and spatial dimensions, and then the attention maps are multiplied by the input feature maps to perform adaptive feature refinement. In 2017, the Google team [29] proposed a transformer that completely relied on the self-attention mechanism to replace the convolutional neural network. The model is widely used in the field of natural language processing due to its efficient parallelism and faster training speed. Liu et al. [30] successfully applied the transformer model to the field of object detection and proposed the Swin-transformer backbone network. The framework extracts image feature information through a convolution-like moving window mechanism (shifted windows), which has certain modeling flexibility at different scales and has a linear computational complexity related to image size. However, datasets of a huge amount of data are needed to optimize transformer models, which hinder the application in many fields.

## 3. Methodology

### 3.1. YOLOv4 Object Detection Model

As a classic in the YOLO series, YOLOv4 has excellent overall detection performance, which is widely used in vehicle–pedestrian detection in traffic scenes. The YOLOv4 network model consists of three major parts: backbone, neck, and head.

**Figure 1:**
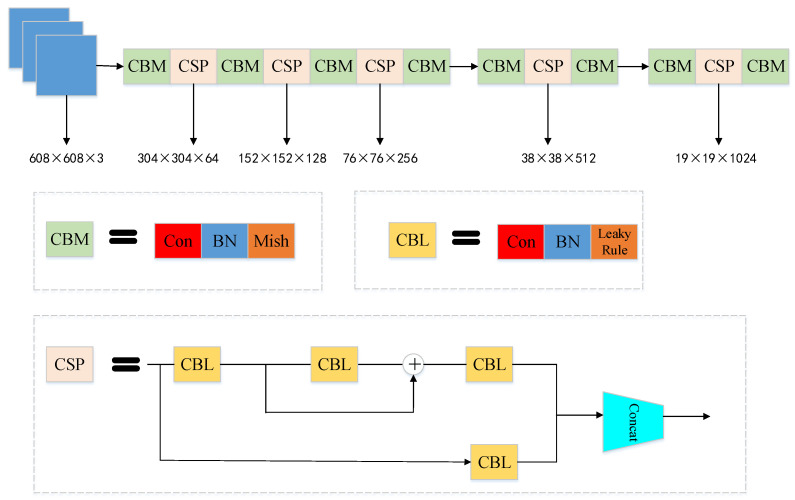
The network structure of CSPDarknet53.

The backbone network is the CSPDarknet53 feature extraction network, which stacks five CSP modules to extract the feature information of images greatly. Figure 1 is the structure of CSPDarknet53.The CSP module is the fusion of a large residual structure and a small residual structure. It contacts the input directly, and the convolution results to form the new output, which can effectively improve the learning ability of the network by reducing repeated gradient learning. The CBM and CBL are a combination of convolution, standardization, and activation function. After the CSPDarknet53 backbone, three efficient feature layers can be obtained.

The neck network includes Spatial Pyramid Pooling (SPP) and PANet. The SPP structure processes the obtained feature layer by max-pooling operation at different scales of 5 × 5, 9 × 9 and 13 × 13, which preserves the image feature information to the greatest extent, increases the receptive field, and suppresses the overfitting of the model effectively. Then, three feature layers are passed through PANet, which achieve the fusion of a semantic feature and a location feature.

The head introduces multi-scale detection logic and multi-label classification ideas to accomplish prediction. Simultaneously, the loss function of YOLOv4 is optimized, which includes location loss, confidence loss, and classification loss. The formula of the loss function can be calculated as

(1) loss=location_loss+confidence_loss+class_loss

(2) $$location\_loss=\lambda_{\mathrm{coord}\;}\sum_{i=0}^{K\times K}\sum_{j=0}^{M}I_{ij}^{obj}\left(2-w_{i}\times h_{i}\right)$$

(3) $$\begin{array}{l} confidence\_loss=\sum_{i=0}^{K\times K}\sum_{j=0}^{M}I_{ij}^{obj}\left[{\hat{C}}_{i}\log\left(C_{i}\right)+\left(1-{\hat{C}}_{i}\right)\log\left(1-C_{i}\right)\right]\\ +\lambda_{\mathrm{noobj}\;}\sum_{i=0}^{K\times K}\sum_{j=0}^{M}I_{ij}^{\mathrm{noobj}\;}\left[{\hat{C}}_{i}\log\left(C_{i}\right)+\left(1-{\hat{C}}_{i}\right)\log\left(1-C_{i}\right)\right] \end{array}$$

(4) $$class\_loss=\sum_{i=0}^{K\times K}I_{ij}^{obj}\sum_{c\in \;\mathrm{classes}\;}\left[{\hat{p}}_{i}(c)\log\left(p_{i}(c)\right)+\left(1-{\hat{p}}_{i}(c)\right)\log\left(1-p_{i}(c)\right)\right]$$

where *λ_coord_* and *λ_noobj_* are penalty coefficients, *K* is the size of grid, *I* is the *i*-th square of the feature map, *j* is the *j*-th prediction box predicted by the square, *w* and *h* represent the width and height of the ground truth. ${\hat{C}}_{i}$ and $C_{i}$ represent the categories of prediction and actual boxes, respectively, ${\hat{p}}_{i}$ is the confidence of the predicted value, $p_{i}$ is the confidence of the actual value. $I_{ij}^{obj}$ and $I_{ij}^{\mathrm{noobj}\;}$ represent the presence and absence of object in the *j*-th anchor box of the *i*-th grid.

In the decoding stage, each grid is used to calculate the position of the prediction box, combined with the corresponding offsets and the size of the anchor box. The position of the prediction box is shown in formula.

(5) $$\left({\hat{x}}_{i}+x\_\mathrm{offset}-\frac{{\hat{\omega }}_{i}}{2},{\hat{y}}_{i}+y\_\mathrm{offset}-\frac{{\hat{h}}_{i}}{2},{\hat{x}}_{i}+x\_\mathrm{offset}+\frac{{\hat{\omega }}_{i}}{2},{\hat{y}}_{i}+y\_\mathrm{offset}+\frac{{\hat{h}}_{i}}{2}\right)$$

where x_offset and y_offset are prediction center offsets of in the horizontal and vertical directions. $({\overset{\wedge }{w}}_{i},\overset{\wedge }{h_{i}})={\overset{\wedge }{s}}_{\overset{\wedge }{x_{i}},\overset{\wedge }{y_{i}}}$ is the ratio of height and width.

### 3.2. MobielNetv2-YOLOv4

MobileNetv2 [31,32] is a lightweight backbone network proposed by Google, which is widely used in mobile and embedded devices.

**Figure 2:**
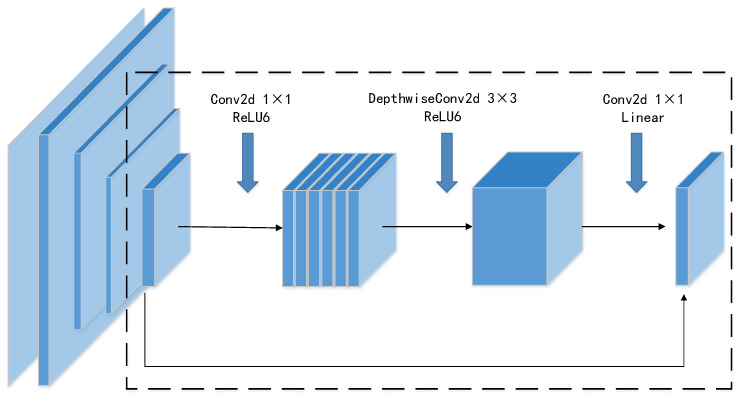
The network structure of Invered_Res_block.

As shown in Figure 2, the main structure of MobileNetv2 is that the Invered_Res_block, which is applied with depthwise separable convolutional blocks, is used to reduce greatly the number of parameters and calculations at a small cost of accuracy.

Compared with the traditional convolutional neural structure, the main characteristics of the MobileNetv2 are as follows. The model uses 1 × 1 convolution to expand the dimension of feature maps, then extracts features by the 3 × 3 depthwise separated convolution, and finally reduces channels by 1 × 1 convolution. Simultaneously, the ReLU6 activation function is used to replace ReLU, which effectively suppresses the maximum value to prevent the decline of accuracy caused by unrestricted output.

**Figure 3:**
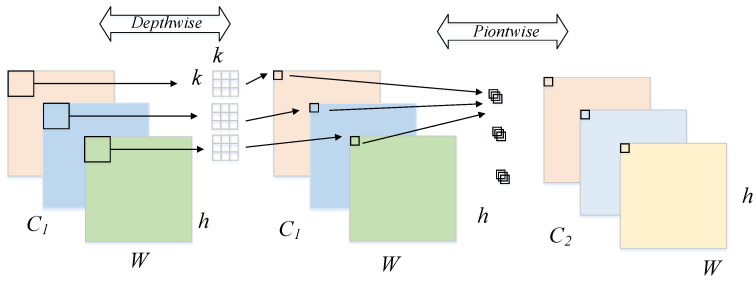
The process of Depthwise Separable Convolution.

Depthwise Separable Convolution (DSC) includes two parts: depthwise convolution and pointwise convolution, as shown in Figure 3. The former is to obtain the spatial information of feature maps on each channel, and the latter is to associate the above channel features information by a standard convolution. Due to the great number of convolutions with a size of 3 × 3 in MobileNetv2, the parameters and calculation costs of the model are greatly reduced. The parameter and calculation comparison between DSC and standard convolution can be shown as

(6) $$\frac{P_{DSC}}{P_{CONV}}=\frac{D_{input}^{2}D_{DW}^{2}M+D_{input}^{2}MN}{D_{input}^{2}D_{DW}^{2}MN}=\frac{1}{N}+\frac{1}{D_{DW}^{2}}$$

where $P_{DSC}$, $P_{CONV}$ are the computation amount of depthwise separable convolution and standard convolution, $D_{input}^{}$, N are the size of the input feature and the number of channels, $D_{DW}^{}$ is the size of the convolution kernel of the depthwise convolution, and M is the number of channels of the pointwise convolution.

The YOLOv4 algorithm is widely used in vehicle and pedestrian detection due to its excellent detection performance, but it also has complex network structures with a large number of computing parameters, which is difficult in deployed in embedded devices. Inspired by the MobileNetv2 [33], the improved model in this paper changes the backbone from CSPdarknet53 to MobileNetv2 to reduce the number of model parameters. Meanwhile, the Spatial Pyramid Pooling (SPP) is added into MobileNetv2, which enhances the receptive field greatly. It can improve the scale invariance and suppresses the overfitting of the model effectively.

### 3.3. Improved Enhanced Feature Extraction Network

**Figure 4:**
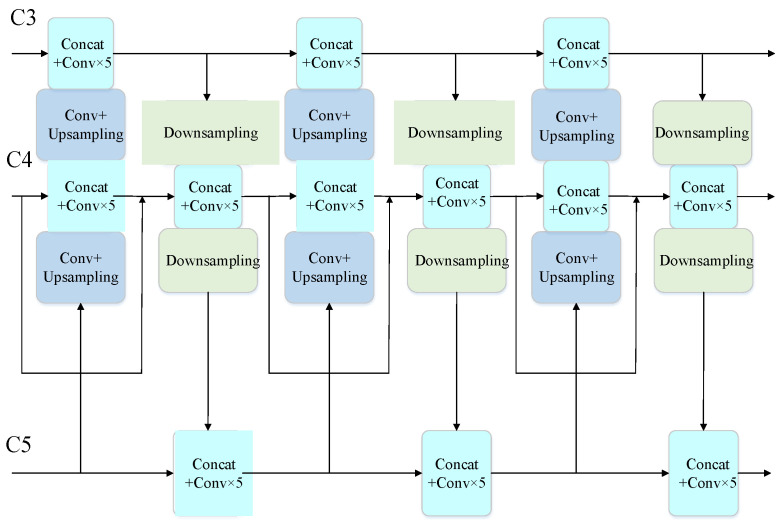
The structure of enhanced feature network BiFPN.

With the further development of the convolutional neural network, it is difficult to retain enough features at high resolution. The low-resolution feature map contains more detailed location information, but the high-resolution feature map contains richer semantic information. Therefore, the effective fusion of shallow features and deep features is crucial to ensure the detection of tiny and diverse targets in traffic scenes. Feature Pyramid Network (FPN) [34], proposed by Lin et al., is designed to fuse different feature maps of all scales via top-down and lateral connections. Inspired by the idea of bidirectional cross-scale fusion in BiFPN proposed by Google, the model proposed in this paper adjusts the feature channels multiple times to concatenate feature information of adjacent feature layers. The connections between input nodes and output nodes of the same level across layers shorten the path from low-level layers to high-level layers. Simultaneously, lateral connections are added between features of the same scale to alleviate the problem of feature loss caused by network deepening. In Figure 4, two feature maps output from the backbone and one feature map output from the SPP are integrated to enrich the semantic information of features in BiFPN. Then three effective feature maps of (52, 52, 128), (26, 26, 256), (13, 13, 512) generated by the multi-scale fusion network are fed into Yolo Head.

### 3.4. CA-MobileNetv2-YOLOv4

**Figure 5:**
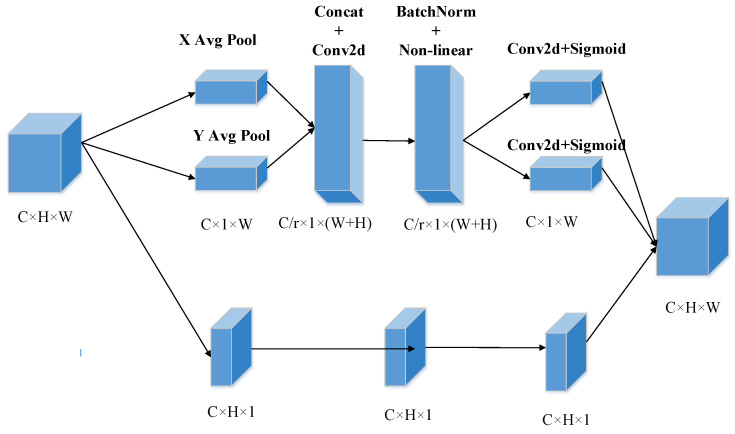
The network structure of coordinate attention mechanism.

Attention Mechanism is to simulate the mechanism by which humans can naturally and effectively discover salient regions in complex scenes. In the field of computer vision, it is a dynamic weight adjustment process based on input image features [35]. Coordinate attention (CA) mechanism [36] is a novel, lightweight and efficient attention mechanism that can be easily integrated into the network and improve the feature extraction ability of the detection network with less extra computational cost. The coordinate attention module is regarded as a computational unit and aims to enhance the expressiveness of the learned functions. It can generate an output tensor *Y = [y1, y2,……, yc]∈R^C^^×H^^×W^* of the enhanced feature representation based on any intermediate tensor *X = [x1, x2, ……, xc]∈R^C^^×H^^×W^*. The CA Block module is mainly divided into two stages, i.e., coordinate information embedding and coordinate attention generation. The former replaces the global pooling method in the traditional channel attention mechanism by encoding 1D features along the 2D direction of the feature map, and the latter captures precise location information by enabling the global receptive field and generates weight values. The CA block is shown in Figure 5.

Global pooling is often used to decode spatial information by compressing global spatial information into feature channels directly, which causes the loss of coordinate information. In the information embedding stage, aiming to achieve long-range interaction with precise location information in the spatial dimension, the 2D global pooling of channel attention is decomposed into two 1D global pooling in the horizontal and vertical directions separately. The decomposition can be calculated as

(7) $$z_{c}^{h}(h)=\frac{1}{W}\underset{0\leq i<W}{\sum }x_{c}(h,i)$$

(8) $$z_{c}^{w}(w)=\frac{1}{H}\underset{0\leq j<H}{\sum }x_{c}(j,w)$$

where $z_{c}^{h}(h)$, $z_{c}^{w}(w)$ are the outputs of the *c*-th channel at height and width, and $x_{c}(h,i)$, $x_{c}(j,w)$ are the inputs of model.

Compared with the mechanism of channel attention that uses global average pooling in encoding all pixels in each channel, the above transformations aggregate and encode each channel along the horizontal and vertical coordinates to generate a pair of orientation-aware feature maps. The coordinate attention module captures long-range correlations in one spatial coordinate direction while retaining precise location information in the other direction, which helps the network locate important features more precisely.

In the coordinate attention generation stage, the feature maps produced by Equations (2) and (3) are aggregated, concatenated, and sent to the shared 1 × 1 convolution transformation function. Then, the intermediate feature maps are produced through a nonlinear activation function, which can be written as

(9) $$f=ReLU\left(Com_{1\times 1}\left(concat\left(z^{\prime \prime },z^{w}\right)\right)\right)$$

where $f\in R^{C/r\times (H+W)}$ is the intermediate feature representation of the spatial feature in the $f^{h}$ and $f^{w}$ directions, *r* represents the down sampling ratio, $\left(concat\left(z^{\prime \prime },z^{w}\right)\right)$ is the concatenated operation along the spatial dimension. These two independent tensors $f^{h}\in R^{C/r\times H}$, $f^{w}\in R^{C/r\times W}$ are divided from $f\in R^{C/r\times (H+W)}$ along the spatial dimension. These two 1 × 1 convolutions $F^{h}$, $F^{w}$ are used to unite the channels.

(10) $$g^{h}=\sigma \left(F_{h}\left(f^{h}\right)\right)$$

(11) $$g^{w}=\sigma \left(F_{w}\left(f^{w}\right)\right)$$

where σ is the sigmoid function. The attention weight values are produced by expanding $g^{h}$, $g^{w}$ partly. The final feature output of the CA module is represented as

(12) $$y_{c}(i,j)=x_{c}(i,j)\times g_{c}^{b}(i)\times g_{c}^{w}(j)$$

**Figure 6:**
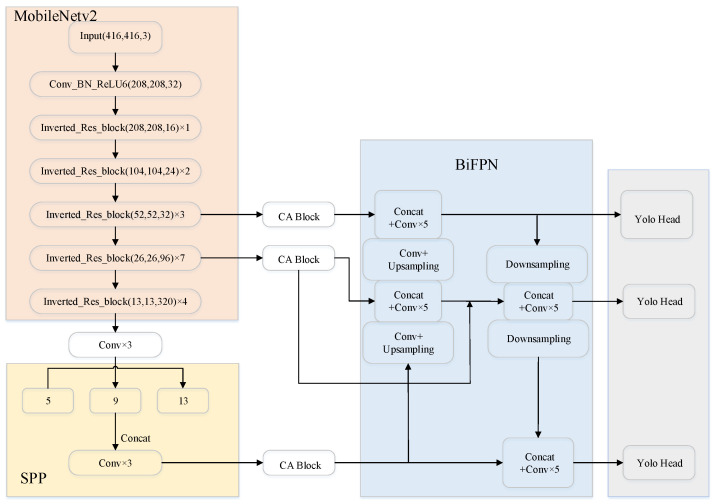
Model structure of CA-MobileNetv2-YOLOv4.

Figure 6 shows the network structure of the improved detection model proposed in this paper. It can be seen that a coordinate attention mechanism is embedded to improve detection capability for traffic targets and to make it suitable for vehicle–pedestrian detection scenarios.

## 4. Experimental Results and Analysis

### 4.1. Experimental Environment and Parameter Description

The CA-MobielNetv2-YOLOv4 proposed in this paper for the traffic scene requires a large number of computing tasks in the training process, and it is difficult for a pure CPU to meet the requirements. Therefore, the model described in this paper is all calculated on high-performance GPU. All experiments have been conducted on the platform configured Win10 operation system, equipped with Intel© Core© i7-10700F 2.90 GHz and GeForce RTX3070 with 8 GB memory. The parallel computation is Cuda 11.2, and the deep neural network acceleration library is cudnn 8.1.0. The utilized deep learning framework is based on Pytorch (GPU) and Python 3.8.

**Figure 7:**
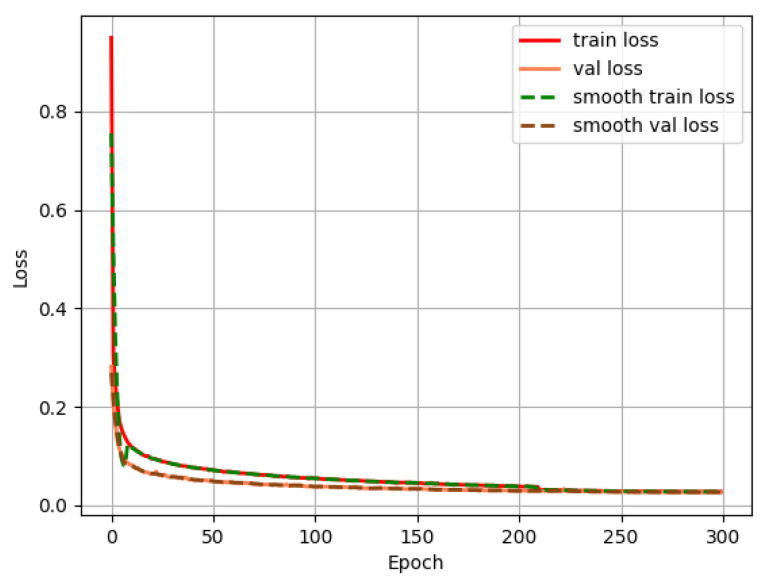
Loss convergence curve of CA-MobileNetv2-YOLOv4.

In the training process, the image input size, the batch size, and the epoch are set as 416 × 416, 16, and 300, respectively. After that, the initial learning rate is set as 0.01, but cosine annealing is used to reduce the learning rate from 0.01 to 0.0001. The loss value is noted for each epoch, and the loss convergence curve of the above-improved YOLOv4 is shown in Figure 7.

The loss convergence curve indicates that the train loss and validation loss continue to decline and eventually converge to the minimum. There is no existence of diverge and overfit, which shows the effectiveness of the improved model of YOLOv4.

### 4.2. Datasets

The PASCAL VOC dataset is a universal dataset in the object detection field, which includes 20 categories of car, person, bus, bicycle, airplane, etc. BD100K is a large-scale and diverse autonomous driving dataset, including road target detection, lane detection, driving area detection, and so on. It selected 10,000 images as the dataset, including 8100 train set, 900 validation set, 1000 test set. KITTI is one of the professional datasets in the field of autonomous driving, including vehicle detection, vehicle tracking, and semantic segmentation. KITTI is currently the largest evaluation dataset for autonomous scenarios in the world. It contains real images collected in urban, rural, and highway scenes, with up to 15 vehicles and 30 pedestrians in each image, with various degrees of occlusion and truncation. It selected 7481 2D images as the dataset, including 6058 train set, 674 validation set, 749 test set. Meanwhile, all the models in this paper are verified in the real experimental dataset, which is called “Ours” in the following content. This paper adjusts the original dataset format to our desired data format, xml, to satisfy our detection model.

### 4.3. Evaluation Metrics

In this paper, the above-mentioned optimized network is accurately compared by analyzing *Precision*, *Recall*, *AP*, et al. The formula is as follows.

(13) $$Precision=\frac{TP}{TP+FP}$$

(14) $$Recall=\frac{TP}{TP+FN}$$

where *TP*, *TF*, and *FN* denote true positive, false positive, and false negative, respectively. *AP* and *mAP* are commonly used as evaluation metrics in the actual engineering field of object detection, which can comprehensively reflect the performance of the model. The value of AP is calculated from the area formed between the *Precision, Recall*, and the horizontal and vertical axes. The value of *mAP* represents the average of all *AP* values, which is shown in the formula.

(15) $$AP=\overset{n-1}{\underset{i=1}{\sum }}\left(r_{i+1}-r_{i}\right)P_{\mathrm{inter}}\left(r_{i}+1\right)$$

(16) $$mAP=\frac{\sum_{i=1}^{k}AP_{i}}{k}$$

### 4.4. Experimental Results

The above metrics are used to evaluate the improved object detection network, as shown in the table above.

As is shown in Table 1, where “✓” represents the corresponding method in each model, it can be seen that the parameters of improved model 2 are 3 far less than the original one, which is reduced by about 85%. Simultaneously, there is little difference in *mAP* value between Model 1 and Model 2. It demonstrates that the backbone of MobileNetv2 shows great lightweight performance without sacrificing detection accuracy. In terms of detection speed, the FPS of Model 1 is 30, the FPS of Model 2 is 38, the FPS of Model 3 is 37, and the FPS of Model 4 is 35. Compared with Model 1 without a lightweight structure, others that optimized with MobileNetv2 have a significant value improvement in FPS. To sum up, the application of the lightweight structure is indeed beneficial to reduce the number of parameters and improve the detection speed.

Compared with Model 2, Model 3 has an increase of 1.31% in *mAP*, which indicates the effectiveness of BiFPN in improving detection performance. Although Model 3 increases the number of parameters, it is still much lower than Model 1. Meanwhile, compared with Model 3, there is a 5% increase in parameters and a 0.5% increase in *mAP* in Model 4, although it sacrifices detection speed to some extent. To sum up, the method of multi-feature fusion, namely BiFPN, does improve the detection accuracy of the model, and the application of the CA is indeed beneficial to improve detection accuracy with little computation cost.

**Figure 8:**
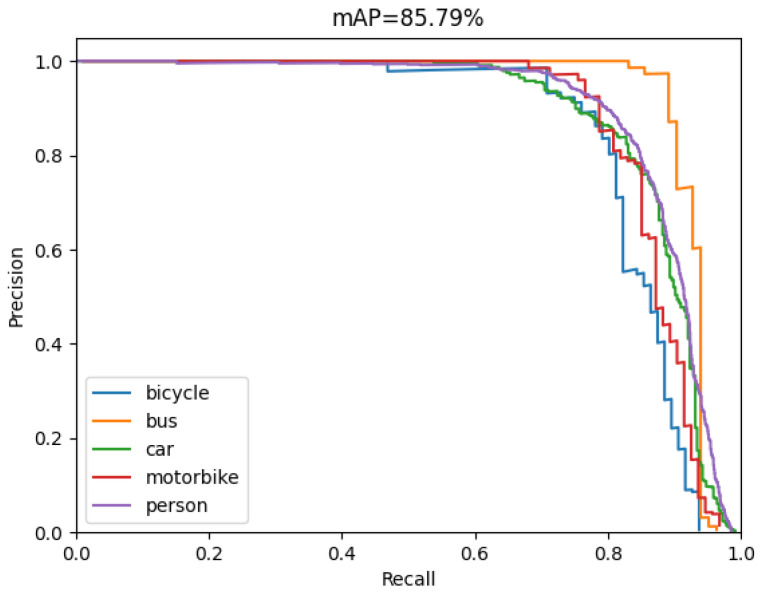
The *mAP* of Model 4 in the VOC dataset.

In the project, the P-R curve is the evaluation curve of detection performance generally. Figure 8 shows the P-R curves of Model 4 on the PASCAL VOC dataset, which include five categories of bicycle, bus, car, motorbike, and person.

**Figure 9:**
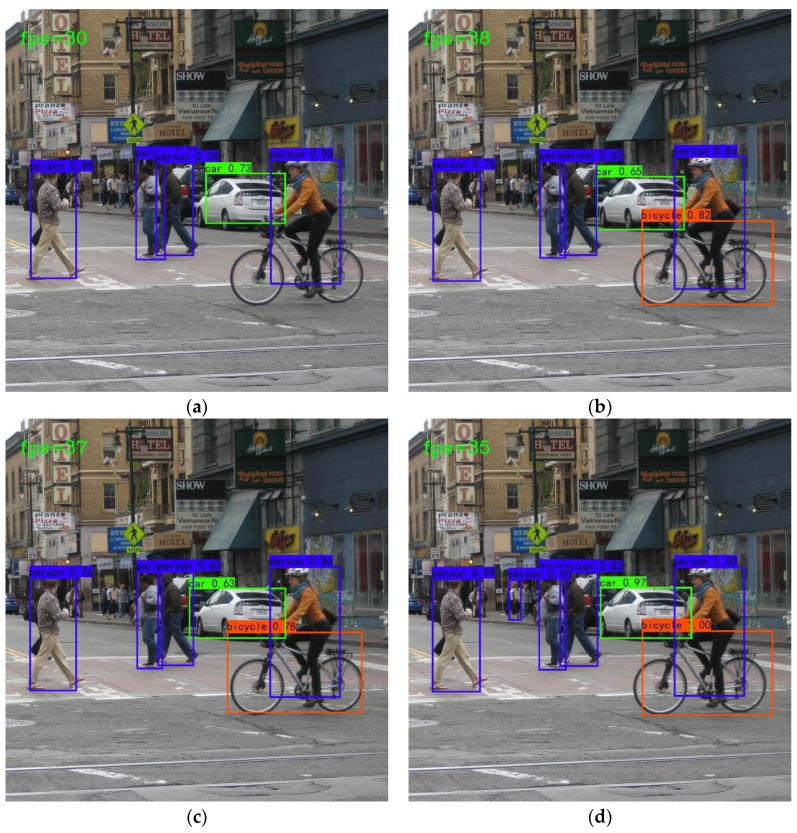
Detection Examples of the above model. The above four pictures are the detection results of the four models, respectively. (**a**) is result of Model 1, (**b**) is result of Model 2, (**c**) is result of Model 3, and (**d**) is result of Model 4.

Figure 9 shows image prediction examples of the above models. Compared with others, Model 4 has the highest detection accuracy, and almost all detected object confidences are close to 1. In Figure 9a, the confidences of the detected target are 0.89, 0.58, 0.73 0.90, and the bicycle is missed detection. In Figure 9d, the confidences of the detected object are 0.99, 0.82, 0.97, 1.00, and the missed target is detected.

**Figure 10:**
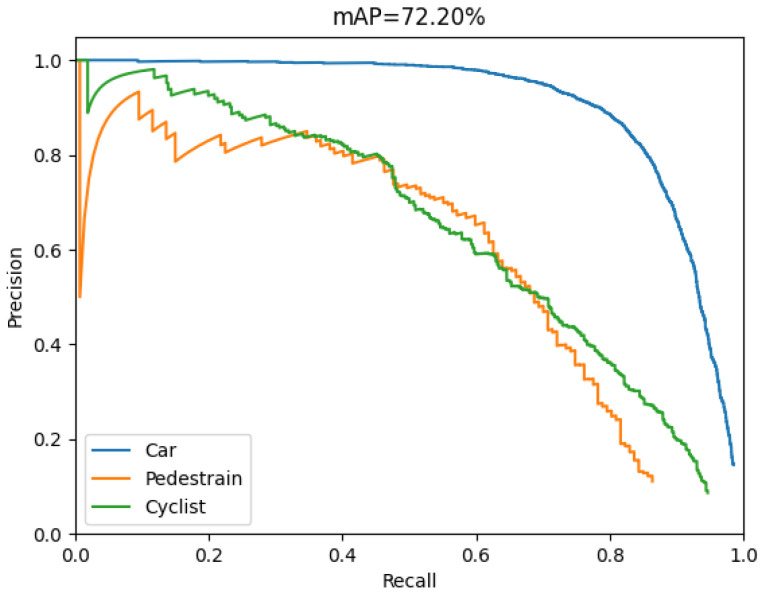
The *mAP* of the Model 4 in the KITTI dataset.

According to the above comparison results, Model 4 maintains the great balance between detection accuracy and speed, compared with others. Therefore, the Model 4 is tested and verified on the KITTI dataset to realize the vehicle–pedestrian detection in traffic scenarios. Figure 10 shows the recall and precision curves of Model 4 on KITTI dataset. As shown in the Figure 10, the *mAP* of Model 4 is up to 72.20%, which indicates that Model 4 is very sensitive to vehicle–pedestrian detection.

**Figure 11:**
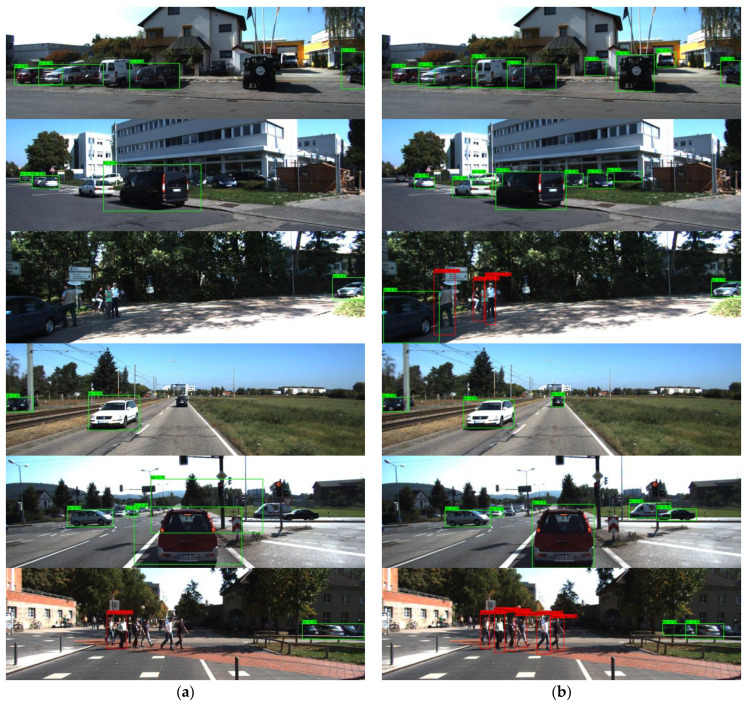
The visualization of vehicle–pedestrian detection results in the traffic scene. (**a**) are the detection results of the original YOLOv4, (**b**) are the results of the improved YOLOv4 (Model 4).

Figure 11 shows the visualization comparison of Model 4 in traffic scenes. In Figure 11a, there are many false and missed detection of a vehicle or pedestrian. However, in Figure 11b, most targets in each traffic scene are detected, which indicates that the improved YOLOv4 algorithm performs very well in vehicle–pedestrian detection in autonomous driving scenarios.

### 4.5. Comparison Detection Performacne on Differernt Datasets

According to the above comparison results, more experiments are presented to test better the effectiveness of the model in traffic scenarios. The experimental results on different datasets are shown in Table 2.

From Table 2, the values of *mAP* and FPS are tested on KITTI, BDD100K, and Ours. Compared with Model 1, Model 2, and Model 3, the *mAP* of Model 4 has obvious improvement that there is about a 2.1% increase on KITTI, a 2.3% increase on BDD100K, and a 2.5% increase on our own dataset. Although there is a small reduction of Model 4 in detection speed due to model complexity, Model 4 reaches a better balance between detection accuracy and detection speed.

**Figure 12:**
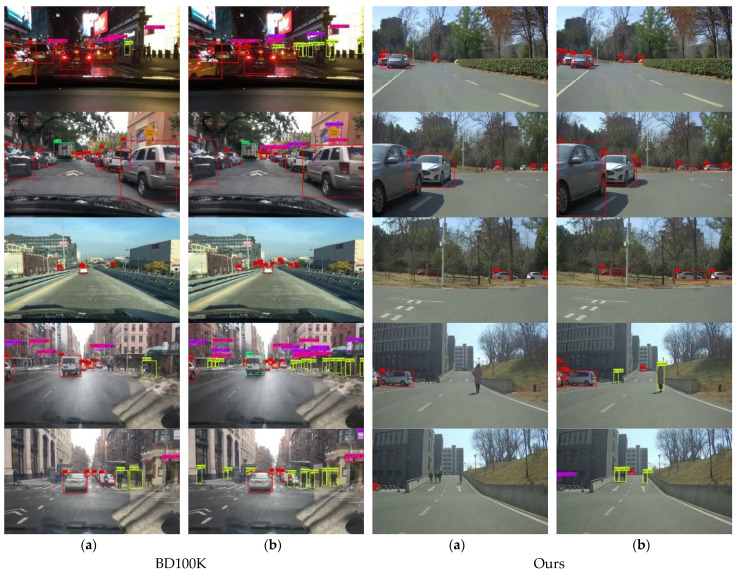
The visualization of vehicle–pedestrian detection results on BD100K and Ours. (**a**) Are the detection results of the original YOLOv4, (**b**) are the detection results of the improved YOLOv4 (Model 4).

In aiming to visualize better the detection performance of the improved YOLOv4, pictures of BD100K and experimental scenarios were predicted, respectively, and the comparison results are shown in Figure 12. Through the results of visualization, it is not difficult to find that most of the targets in the picture are effectively detected by optimizing the YOLOv4 model.

### 4.6. Comparison of Detection Performance with Other Algorithm

In aiming to verify further the effectiveness of the algorithm in this paper, the proposed CA-MobileNetv2-YOLOv4 is compared with the current mainstream one-stage object detection algorithm. The comparison results are shown in Table 3.

It can be seen from Table 3 that the *mAP* and FPS of CA-MobileNetv2-YOLOv4 proposed in this paper are greatly improved, and the parameters are much lower than the above algorithm. Compared with YOLOv3, the CA-MobileNetv2-YOLOv4 is 6.94% higher on *mAP* and 16.7% higher on FPS. Compared with YOLOv4, the CA-MobileNetv2-YOLOv4 is 5.4% higher on *mAP* and 25% higher on FPS. Although the CA-MobileNetv2-YOLOv4 is 1% lower than YOLOv5 on *mAP*, it has an increase of 34.6% on FPS. Similarly, the huge number of parameters of YOLOv5 makes it not meet the requirements of lightweight, which is not conducive to the deployment. In general, the CA-MobileNetv2-YOLOv4 is more cost-effective, trades off detection speed and accuracy to a certain extent, and is more suitable for vehicle–pedestrian detection in traffic scenarios.

## 5. Conclusions

This paper proposes a lightweight and high-performance vehicle detection algorithm based on YOLOv4, namely CA-BiFPN-MobileNetv2-YOLOv4, to solve the problems caused by complex environmental factors in traffic scenes. Specifically, it utilizes a lightweight backbone network for image feature extraction, then introduces the coordinate attention mechanism to capture long-term dependencies with precise location information to fully focus on useful features of images and use BiFPN for feature fusion to sufficiently merge the high-level semantic information and low-level details. Additionally, the experimental results of the model on the PASCAL VOC datasets indicate that the improved model’s is up to 85.79% and FPS is up to 35, which has significant performance promotion, compared with the 81.08% and 30 FPS of the original YOLOv4. Furthermore, the improved YOLOv4 model maintains a great balance between detection accuracy and speed on different datasets, indicating that it can be applied to object detection in traffic scenarios. In the future, more advanced algorithms applied to vehicle–pedestrian detection can be conducted in-depth research to improve the overall performance and practical application value of the algorithm.

## Figures and Tables

**Table 1:** Model performance comparison.

	MobileNetv2	BiFPN	Coordinate Attention	Parameters (M)	*mAP* (%)	FPS (Hz)
Model 1				64.62	81.08	30
Model 2	✓			11.15	83.98	38
Model 3	✓	✓		20.16	85.29	37
Model 4	✓	✓	✓	21.16	85.79	35

**Table 2:** Experimental comparison on different datasets.

	KITTI		BDD100K		Ours	
	*mAP* (%)	FPS (Hz)	*mAP* (%)	FPS (Hz)	*mAP* (%)	FPS (Hz)
Model 1	67.9	29	73.8	30	70.1	30
Model 2	69.3	37	75.1	40	72.0	39
Model 3	70.1	36	76.0	38	72.7	37
Model 4	72.2	35	78.3	37	75.2	36

**Table 3:** Comparison results of other algorithms on KITTI datasets.

Method	Backbone	Input Size	Parameters (M)	*mAP* (%)	FPS (Hz)
YOLOv3	Darknet53	640 × 640	58.65	65.26	30
YOLOv4	CSPDarknet53	640 × 640	64.62	66.80	28
YOLOv5	CSPDarknet53	640 × 640	86.70	73.20	26
CA-MobileNetv2-YOLOv4 (Ours)	MobileNetv2	640 × 640	21.16	72.20	35

## Data Availability

Not applicable.
